# Environmental Epigenetics and Genome Flexibility: Focus on 5-Hydroxymethylcytosine

**DOI:** 10.3390/ijms21093223

**Published:** 2020-05-02

**Authors:** Olga A. Efimova, Alla S. Koltsova, Mikhail I. Krapivin, Andrei V. Tikhonov, Anna A. Pendina

**Affiliations:** D. O. Ott Research Institute of Obstetrics, Gynecology and Reproductology, Mendeleevskaya line 3, 199034 St. Petersburg, Russia; rosenrot15@yandex.ru (A.S.K.); krapivin-mihail@mail.ru (M.I.K.); tixonov5790@gmail.com (A.V.T.); pendina@mail.ru (A.A.P.)

**Keywords:** 5-hydroxymethylcytosine, DNA methylation, environmental factors, phenobarbital, narcotics, dimethyl sulfoxide (DMSO), heavy metals, bisphenol A (BPA), particulate air pollution, pentachlorophenol (PCP)

## Abstract

Convincing evidence accumulated over the last decades demonstrates the crucial role of epigenetic modifications for mammalian genome regulation and its flexibility. DNA methylation and demethylation is a key mechanism of genome programming and reprogramming. During ontogenesis, the DNA methylome undergoes both programmed changes and those induced by environmental and endogenous factors. The former enable accurate activation of developmental programs; the latter drive epigenetic responses to factors that directly or indirectly affect epigenetic biochemistry leading to alterations in genome regulation and mediating organism response to environmental transformations. Adverse environmental exposure can induce aberrant DNA methylation changes conducive to genetic dysfunction and, eventually, various pathologies. In recent years, evidence was derived that apart from 5-methylcytosine, the DNA methylation/demethylation cycle includes three other oxidative derivatives of cytosine—5-hydroxymethylcytosine (5hmC), 5-formylcytosine, and 5-carboxylcytosine. 5hmC is a predominantly stable form and serves as both an intermediate product of active DNA demethylation and an essential hallmark of epigenetic gene regulation. This makes 5hmC a potential contributor to epigenetically mediated responses to environmental factors. In this state-of-the-art review, we consolidate the latest findings on environmentally induced adverse effects on 5hmC patterns in mammalian genomes. Types of environmental exposure under consideration include hypnotic drugs and medicines (i.e., phenobarbital, diethylstilbestrol, cocaine, methamphetamine, ethanol, dimethyl sulfoxide), as well as anthropogenic pollutants (i.e., heavy metals, particulate air pollution, bisphenol A, hydroquinone, and pentachlorophenol metabolites). We put a special focus on the discussion of molecular mechanisms underlying environmentally induced alterations in DNA hydroxymethylation patterns and their impact on genetic dysfunction. We conclude that DNA hydroxymethylation is a sensitive biosensor for many harmful environmental factors each of which specifically targets 5hmC in different organs, cell types, and DNA sequences and induces its changes through a specific metabolic pathway. The associated transcriptional changes suggest that environmentally induced 5hmC alterations play a role in epigenetically mediated genome flexibility. We believe that knowledge accumulated in this review together with further studies will provide a solid basis for new approaches to epigenetic therapy and chemoprevention of environmentally induced epigenetic toxicity involving 5hmC patterns.

## 1. Introduction

In 1972, a group of investigators observed the presence of modified cytosine, 5-hydroxymethylcytosine (5hmC), which accounted for about 15% of the total cytosine residues in rat and mouse brain DNA [1]. Further attempts to reproduce these results failed for a long time, the biological role of 5hmC remaining unknown for roughly the next 40 years. In 2009, however, 5hmC was repeatedly detected in mouse brain cells [2] and mouse embryonic stem cells (mESCs) [3]. Moreover, 5hmC was identified as the oxidation product of methylated cytosine, 5-methylcytosine (5mC) [3], which is a major player in epigenetic regulation and genome reprogramming in mammalian development [4,5,6,7]. 

5mC oxidation is mediated by the enzymatic activity of TET (Ten-Eleven-Translocation) family proteins and consistently yields three oxidative derivatives: 5-hydroxymethylcytosine, 5-formylcytosine (5fC), and 5-carboxylcytosine (5caC). Once targeted by base excision repair enzymes, 5fC and 5caC are excised from the DNA and replaced with unmodified cytosine [3,8]. TET-mediated 5mC oxidation apparently promotes active (enzymatic) DNA demethylation which is a key event of epigenetic reprogramming in germ cells and mammalian embryos.

Research demonstrated that 5mC oxidation products serve as intermediates in active DNA demethylation and likewise function in genome regulation. This is particularly true for 5hmC as a predominantly stable oxidation product of 5mC. Thus, 5hmC is specifically recognized by some protein regulators of cell metabolism, including RPL26, PRP8, MHS6, MeCP2, UHRF, and Thy28 [9,10,11]. Typically, 5hmC would exhibit specific genomic localization—in enhancers, sites flanking promoters (or CpG-islands), and in gene bodies. Furthermore, abundance of 5hmC at enhancers is positively correlated with enhancer activity [12,13,14]. At CpG-islands, 5hmC is vital to maintain promoters in the unmethylated state, whereas in intragenic sequences, 5hmC is suggested to have an inhibitory action on antisense transcription initiation [15,16]. 5hmC can be thus recognized as a stable cytosine modification which has its own function [17,18,19,20,21].

On the one hand, in the course of ontogenesis, specific epigenetic profiles that act to initiate gene expression programs and direct cell differentiation are established, maintained, and altered in a strictly determined way [22,23,24]. Reversible cytosine modification, on the other hand, provides a source of epigenomic plasticity—the ability of the epigenome to change in response to external factors. Today, there is well-established evidence that aberrant DNA methylation patterns generally associated with some pathological conditions including genome structural variation and complex rearrangements can be provoked by endogenous and environmental factors [25,26,27,28,29]. Folic acid deficiency during pregnancy can induce a deficiency of S-adenosylmethionine (SAM), a methyl group donor, resulting in elevated homocysteine and abnormal DNA methylation. This leads to gene deregulation, including abnormal biallelic expression of imprinted genes, which are normally characterized by a monoallelic expression [30]. Metals such as nickel, cadmium, and arsenic perturb DNA methylation patterns and damage epigenetic regulation of proto-oncogenes and oncosuppressors, thus increasing the risk of malignization [31]. Research has shown that disrupted DNA methylation can be induced by synthetic nonsteroidal estrogen—diethylstilbestrol [32,33,34]. Ample studies suggest evidence of DNA methylation damage due to exposure to chromium, mercury, trichloroethylene, dichloroacetic and trichloroacetic acid, bisphenol A (BPA), and many other substances [35,36,37,38,39,40,41,42]. 

While DNA methylation has been extensively investigated, knowledge about how 5hmC patterns change in response to adverse environmental factors is so far scarce [43]. Up-to-date information is focused on brain-related 5hmC changes upon external exposures [44] or has been reviewed in terms of the link between environmental cues and DNA hydroxymethylation, lacking discussion of the underlying molecular mechanisms [45]. The present paper consolidates current evidence on 5hmC pattern alterations induced by environmental factors in different organs, tissues, and cell lines and analyzes how they affect genome function. Along with DNA hydroxymethylation changes, the associated alterations of major components for 5hmC production (5mC, TETs, DNA methylases, co-substrates, and co-factors) are reviewed to place special emphasis on the molecular mechanisms providing epigenetic responses to adverse external effectors. The reviewed data strongly suggest that environmentally induced genome-wide and gene-specific hydroxymethylation alterations are driven by various metabolic pathways due to effector-specific changes in 5hmC biochemistry. The associated transcriptional changes point towards the role of environmentally induced 5hmC alterations in epigenetically mediated genome flexibility. 

## 2. Factors Associated with 5hmC Biochemical Pathways in Mammalian DNA

**Figure 1:**
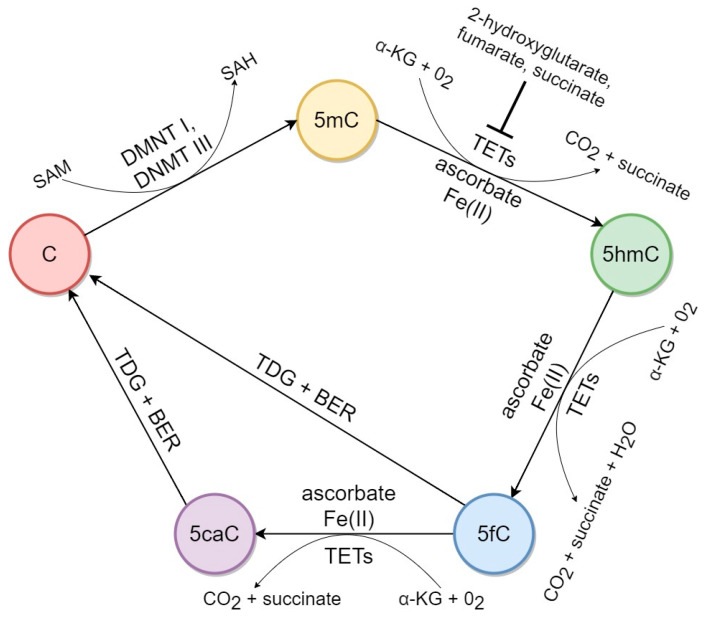
5-hydroxymethylcytosine (5hmC) biochemical pathways in mammalian DNA. 5hmC is produced by TET protein-mediated oxidation (hydroxylation) of 5-methylcytosine (5mC). TET proteins utilize alpha-ketoglutarate (α-KG) and oxygen (O_2_) as co-substrates and require co-factors Fe(II) and ascorbate to yield 5hmC, CO_2_, and succinate. Succinate, fumarate, and 2-hydroxyglutarate are inhibitors of TET activity. Further TET-driven oxidation of 5hmC consistently produces 5-formylcytosine (5fC) and 5-carboxylcytosine (5caC), which are replaced with cytosine (C) by thymine-DNA glycosylase (TDG)-mediated base excision repair (BER). DNA methyltransferases I and III (DNMTs I and III) transfer methyl group from S-adenosylmethionine (SAM) to cytosine producing 5mC and S-adenosylhomocysteine (SAH).

5hmC biochemical pathways in mammalian DNA are shown in Figure 1. 5hmC in mammalian DNA is the product of 5mC oxidation. Although several studies provided evidence for the production of 5hmC by a random hydroxyl radical attack on 5mC under oxidative stress conditions [46,47,48], basically, hydroxylation of methylated DNA is driven by TET proteins [3]. The TET family consists of three proteins—TET1, TET2, and TET3; all of them are dioxygenases that can catalyze the oxidation of 5mC to produce 5hmC both in vivo and in vitro [8]. Apart from 5mC, TET proteins utilize alpha-ketoglutarate and oxygen as co-substrates and require co-factors Fe(II) and ascorbate to yield the reaction end products: 5hmC, CO_2_, and succinate [3]. It is therefore apparent that the mechanism of reaction depends on TET enzymatic activity, availability of substrate, and co-factors.

The main substrate 5mC is derived from DNA methylation: the transfer of methyl group from SAM to the fifth carbon of cytosine in CpG dinucleotide [49]. DNA methylation is catalyzed by a group of specific enzymes—type I and III DNA methyltransferases (or methylases). Type I DNA methyltransferase (DNMT1) is specific to hemimethylated DNA which is produced after replication and contains a methylated template strand and an unmethylated newly synthesized strand. DNMT1 methylation of the new strand mediates reproduction of the template strand methylation pattern and thus enables its inheritance by daughter cells during divisions. Conversely, type III DNA methyltransferases DNMT3A and DNMT3B are specific to unmethylated DNA and can drive methylation de novo [50]. 

Alpha-ketoglutarate is enzymatically produced from isocitrate. The reaction is catalyzed by isocitrate dehydrogenases IDH1, IDH2, and IDH3 [51]. IDH2 dysfunction in gastric cancer cells results in 5hmC depletion [52]. In melanoma cells, IDH2 down-regulation is also associated with decreased 5hmC levels [53]. The latter is induced by 2-hydroxyglutarate accumulation—an oncometabolite that competitively inhibits TET enzymes [54]. Fumarate and succinate, generally accumulated by cancer cells that are exposed to fumarate hydratase and succinate dehydrogenase deficiency, also show inhibitory action on TET proteins and prevent their binding with alpha-ketoglutarate [55,56]. Conversely, excess of IDH1 and IDH2 facilitates the increase of DNA hydroxymethylation [53,54]. Elevated alpha-ketoglutarate in mouse liver cells induces a surge in 5hmC levels [57].

In different cell types, oxygen can affect 5mC hydroxylation in various ways. Throughout embryogenesis, oxygen gradients differentially regulate TET activity and thus determine cellular differentiation [58]. Under hypoxia, human glioblastoma cells exhibit a decrease in 5hmC levels which is associated with hypermethylation and loss of TET activity [59]. On the contrary, human embryonic stem cells (hESCs) show hypoxia-induced TET up-regulation which leads to elevated DNA hydroxymethylation [60]. Similarly, hypoxic neuroblastoma cells demonstrate elevated 5hmC levels induced by hypoxia-inducible factor (HIF) activation that in turn enhances TET activity [61]. 

Ascorbate (l-ascorbic acid or vitamin C) promotes 5hmC increase in the genome [62]. This effect is neither Fe(II)-dependent nor related to changes in *Tet* or *IDH* expression and subsequent alpha-ketoglutarate production [63]. Ascorbate directly interacts with the catalytic domain of TET proteins, reducing Fe(III) to Fe(II) and enhancing TET-mediated 5mC oxidation [64]. Considering that most, if not all, malignant lesions exhibit decreased 5hmC levels [65], ascorbic acid’s potential as an anti-cancer therapy is currently intensively investigated. Evidence suggests that ascorbate-mediated 5hmC increase in melanoma cells suppresses their metastatic capabilities and detains tumor growth [66]. 

The presence of Fe(II) as another co-factor for 5mC hydroxylation also appears to enhance the activity of TET to generate 5hmC [67]. Mutation-induced modifications at the Fe(II)-binding domain of TET proteins lead to a decrease/loss of enzymatic activity [3].

Overall, 5hmC production in the mammalian genome predominantly depends on the presence and level of major components for 5mC oxidation: 5mC itself, TET enzymes, alpha-ketoglutarate, oxygen, Fe(II), and ascorbate.

## 3. Impact of External Factors on Genomic Hydroxymethylation

### 3.1. Hypnotics and Medications

#### 3.1.1. Phenobarbital

Phenobarbital is a barbituric acid derivative with antiseizure, hypnotic, and sedative properties. In rodents, chronic exposure to phenobarbital demonstrated hepatocancerogenic action [68]; there is no evidence of hepatocancerogenic hazard of phenobarbital in humans. Mice that had been treated with phenobarbital in drinking water for 28 days showed elevated 5hmC levels in promoters of tumor-related genes of liver tissue. Elevated 5hmC is associated with decreased DNA methylation and up-regulation of these genes, suggesting the initiation of active DNA demethylation [69,70]. Prolonged exposure to phenobarbital of up to 91 days significantly promotes DNA hydroxymethylation [70]. Experimental data on phenobarbital-induced hepatocellular adenomas demonstrated changes of hydroxymethylation and gene expression levels, including carcinogenic genes, especially those regulated through the constitutive androstane receptor (CAR) signaling pathways [71]. This evidence suggests that changes in 5hmC levels related to the initiation of active DNA demethylation indicate hepatic cell response to phenobarbital, associated with carcinogenicity and other effects.

#### 3.1.2. Diethylstilbestrol 

Diethylstilbestrol is a synthetic nonsteroidal estrogen that was prescribed to pregnant women until 1971 to support pregnancy and prevent miscarriage or other pregnancy complications. The drug was banned after the American Cancer Society provided evidence of carcinogenicity [72]. In mice, neonatal exposure to diethylstilbestrol induces alterations in histone modification pattern and a significant reduction in *Tet1* expression; this correlates with a decrease in 5hmC levels in adults [73]. Considering these results, the authors assumed that it is diethylstilbestrol-induced epigenetic alterations that are responsible for modifications in female reproductive tract gene expression, infertility, and uterine cancer [73]. First-trimester diethylstilbestrol exposure is associated with an increased risk of benign tumors—uterine leiomyomas [74]. Based on detected 5hmC imbalance in uterine leiomyoma tissue [75] and the dependence of the hydroxymethylation pattern on the hormonal status [76], it can be assumed that diethylstilbestrol-induced benign tumorigenesis also involves alterations in 5hmC.

#### 3.1.3. Cocaine

Cocaine is a highly addictive alkaloid of the shrub *Erythroxylum coca*. Mice receiving cocaine intraperitoneal injections for 14 days showed decreased 5hmC levels in liver cells without any alterations in global DNA methylation; meanwhile in brain cells, hydroxymethylation level remained unchanged [77]. Another research also reports the absence of alterations in global DNA methylation and hydroxymethylation in mouse nucleus accumbens in response to cocaine administration [78]. The authors observed significant down-regulation of *Tet1* mRNA and a concomitant decrease in TET1 protein [78]. A ~40% decrease in *TET1* mRNA was observed in the nucleus accumbens of human cocaine addicts examined postmortem [78]. This evidence suggests that cocaine can induce locus-specific 5hmC alterations, while a greater abundance of *Tet2* and *Tet3* mRNAs, characteristic of nucleus accumbens, can presumably compensate for TET1 decrease [78]. Selective chemical labeling for 5hmC followed by deep sequencing allowed identification of 11511 differentially hydroxymethylated regions, distributed primarily in gene bodies (~55%) and intergenic regions (~34%) [78]. In rat prefrontal cortex exposed to cocaine self-administration, both DNA methylation and hydroxymethylation are decreased within the *Homer2* promoter, a glutamate receptor-related scaffolding protein [79]; all *Tet* genes and *Dnmt3b* are down-regulated while *Dnmt3a* is up-regulated [80]. Combined with the effect on brain structures, there is indirect evidence of cocaine-induced alterations in DNA hydroxymethylation patterns in mouse spermatogenic cells: authors observed decreased *Dnmt3b* and *Tet1* mRNAs and increased *Dnmt3a* and DNA methylation [81].

#### 3.1.4. Methamphetamine

Methamphetamine is a synthetic neurotoxic psychostimulant. Its intake causes increase in TET1 and TET3 protein levels and changes hydroxymethylation levels in rat nucleus accumbens [82]. Increased 5hmC concomitant with a decrease in DNA methylation was detected in *corticotropin-releasing hormone* (*Crh*/*Crf*) gene promoter and at a CpG-rich region within the *arginine vasopressin (Avp)* gene body [82]. On the contrary, promoter sequences of *GluA1 and GluA2 alpha-amino-3-hydroxy-5-methyl-4-isoxazole propionic acid receptor* (*AMPAR*) undergo a decrease in both striatal DNA methylation and hydroxymethylation [83]. These changes, combined with histone modifications [83], seem to suppress striatal glutamate receptor expression, observed in systemic methamphetamine intake. Other genomic regions are also affected by methamphetamine-driven changes in 5hmC. In rats addicted to methamphetamine, nucleus accumbens cells subjected to immunoprecipitation with polyclonal anti-5hmC antibodies followed by next-generation sequencing showed numerous differentially hydroxymethylated regions, predominantly in intergenic sites located on long and short interspersed elements [84].

The available data provides supporting evidence that cocaine- and methamphetamine-driven changes in DNA hydroxymethylation patterns can be a vital factor in promoting drug addiction.

#### 3.1.5. Ethanol

Prenatal exposure to ethanol causes disturbances in normal methylation and hydroxymethylation dynamics of developing mouse hippocampus and cortex [85,86]. Delayed 5mC and 5hmC increase is observed in the neuroepithelial stem cells. In early maturing neurons, ethanol disrupts the timely decrease of DNA methylation and increase of hydroxymethylation [85]. Prenatal exposure to ethanol has prolonged action: after withdrawal, developing gyrus dentatus undergoes significant changes in programmed DNA methylation and hydroxymethylation dynamics during the third trimester of pregnancy [85]. After 8 days of ethanol exposure in vitro, mouse neural stem cells demonstrate an increased global DNA methylation level, whereas the total 5hmC level remains unaffected. After withdrawal, however, the DNA hydroxylation level plummets significantly [87]. In blood samples of alcohol-dependent humans, hydroxymethylation is significantly lower compared to controls. During detoxification, a rise of hydroxymethylation is observed [88]. Chronic alcohol consumption in rats decreases hydroxymethylation levels in liver cells by half [89] and intensifies apoptosis of hepatocytes [90]. This is accompanied by decreasing TET1 levels, while TET2 and TET3 remain unchanged [90]. However, iron supplementation to an alcohol diet prevents changes in DNA hydroxymethylation levels [89]. 

#### 3.1.6. Dimethyl Sulfoxide

Dimethyl sulfoxide (DMSO) is a bipolar aprotic solvent. DMSO has been known for its enhanced permeability and capacity to significantly facilitate transdermal permeation of active substances; therefore, DMSO is extensively used in local cosmetic products and medicines for transdermal delivery of local anti-inflammatory and pain-killing agents. DMSO is also used in cryopreservation of different types of cells. Thaler et al. showed that DMSO exposure induced an increase of both global and gene-specific 5hmC levels in pre-osteoblastic MC3T3-E1 cell line [91]. The authors report that 12 to 24 h after DMSO exposure *Tet* and *Gadd45* genes—the key players in DNA hydroxymethylation and nucleotide excision repair—demonstrated increased expression. There was a concurrent decreased expression of genes related to DNA methylation: *Dnmt1*, *Dnmt3b*, and *Hells* [91]. The *Tet1*-dependent pro-apoptotic gene *Fas* and the early osteoblastic factor *Dlx5* demonstrated expression increase. The aforesaid changes in gene expression are associated with a global and gene-specific increase in hydroxymethylation and concomitant gene-specific loss of DNA methylation at *Fas* and *Dlx5* promoters [91]. By day 5, the DMSO impact on promoter-specific and global methylation/hydroxylation is reduced or reversed [91]. The 3D microtissues of a maturing cardiac model and a mature hepatic model provide indirect evidence of DMSO effect on DNA hydroxymethylation in human cells. Adding DMSO at 0.1% to human 3D cardiac microtissue culture promotes up-regulation of methyltransferases *DNMT1* and *DNMT3A* and down-regulation of *TET1*. Transcriptional changes of DNA methylation writers and erasers are associated with changes of methylation levels in 66,178 regions, where 71% show gain of DNA methylation [92]. In contrast, no deregulation of DNA methylation is observed in DMSO-exposed 3D hepatic microtissue [92]. 

### 3.2. Anthropogenic Pollutants

#### 3.2.1. Heavy Metals

Arsenic is listed among the most dangerous substances in the United Nations Environment Programme guidelines. The International Agency for Research on Cancer includes arsenic in Group 1 ‘Carcinogenic to humans’. Arsenic exposure is associated with cardiovascular diseases, diabetes mellitus, and neurological and reproductive disorders [93]. Once ingested, inorganic arsenic compounds undergo enzymatic methylation, a pathway of detoxification utilizing SAM as the methyl donor. Significant amounts of arsenic induce SAM depletion, loss in global DNA methylation, and aberrant locus-specific hypermethylation in multiple regions including *p53* and *p16* promoters. Disrupted DNA methylation patterns, particularly in proto-oncogenes and onco-suppressor genes, increase the risk of malignization [94]. 

The effect of arsenic on DNA hydroxymethylation has been recently demonstrated in both animal and human models. After 8 weeks of exposure to sodium arsenite dissolved in drinking water, male Sprague-Dawley rats showed elevated DNA hydroxymethylation levels in lungs, heart, spleen, and pancreas, while liver and kidney were unaffected. DNA methylation, however, remained unchanged in these types of organs, except for spleen, where the methylation level was elevated [95]. Exposure to arsenic trioxide in drinking water for 6 months induced a decrease in DNA methylation and hydroxymethylation in hippocampus and cortex, presumably promoted by down-regulation of *Dnmts* and *Tets* expression [96]. These changes driven by oxidative stress cause deregulation of tricarboxylic acid cycle and alpha-ketoglutarate pathway. No concurrent SAM decrease was reported [96]. Tricarboxylic acid cycle deregulation and reduced Tet protein activity associated with loss of 5hmC, 5fC, and 5caC are also observed in mESCs under arsenic exposure [97]. Human embryonic kidney cells (HEK293T) were used to show that arsenite can bind directly to the zinc fingers of Tet proteins, thus causing loss of catalytic activity to catalyze oxidation of 5mC to yield 5hmC, 5fC, and 5caC. A successive decrease in 5hmC and an increase in 5mC depends on arsenite concentration [98]. Arsenic-containing hydrocarbons AsHC 332 and AsHC 360 increase global hydroxymethylation and alter expression of a number of genes, including *FEN1*, *XPA*, and *DNMT3A* in culture of human liver cells HepG2 [99]. In individuals with high urine concentration of dimethylarsinate over years, DNA methylation variation in blood correlates positively with changes in 5hmC levels [100]. Remarkably, a different analysis performed on blood samples of arsenic-exposed individuals demonstrated a positive correlation between arsenic exposure and global 5hmC levels in men and a negative correlation in women. Apparently, it is the plasma total homocysteine level that seems to contribute to this sex difference, as positive correlation is stronger in men with normal plasma total homocysteine, whereas negative correlation is stronger in hyperhomocysteinemic women [101]. 

Sporadic studies investigated the effect of other heavy metals on 5hmC patterns. In mESCs culture, cadmium exposure induces a decrease in TET protein activity and a decrease in 5hmC, 5mC, and 5caC levels, while the 5mC level remains unchanged [97]. Exposure to chromium and antimony has a similar action on mESCs [97]. In the Central Zhejiang Province of China, children who live in the vicinity of waste incinerators and have elevated blood levels of chromium, cadmium, and lead show lower mean serum levels of 5mC and 5hmC and a higher mean level of percent tail DNA than children living in unpolluted areas. There is a sex difference in correlation with heavy metals in blood and epigenetic changes: in boys, chromium in blood is negatively correlated with 5mC, while cadmium in blood is positively correlated with 5mC and 5hmC; in girls, however, chromium in blood alone is negatively correlated with 5mC [102]. Mercury exposure in utero is associated with a decrease in 5hmC genomic content and an increase in the 5mC to 5hmC ratio in cord blood at birth and until the age of 5 years [103]. Nickel inhibits TET-mediated 5mC oxidation in human embryonic lung fibroblasts cell culture (MRC5) and HEK293T cells as well as in mESCs and significantly reduces the global 5hmC level [104]. Lead exposure alters hydroxymethylation patterns in CpG islands in hESCs and in the cord blood of newborns [105]. The epigenetic toxicity of other heavy metals is questionable and requires future in-depth research.

#### 3.2.2. Particulate Air Pollution

The relationship between particulate air pollution—a mixture of particles, ranging in diameter—and compromised health has been well documented; this includes a potential progression of respiratory and neurodevelopmental disorders and neurodegenerative diseases [106,107]. Mechanisms for the neuronal pathology of fine particulate matter (PM_2.5_) involve oxidative stress-mediated neurocytotoxicity and abnormal DNA hydroxymethylation increase at the genome level and in promoters of neural genes, including *MeCP2*, *GRIN1*, *GABRB3*, *NRXN1*, and *NLGN3*, as shown on SH-SY5Y human neuroblastoma cell line [108]. Mice systematically exposed to concentrated ambient PM_2.5_ showed a decrease in global 5hmC levels in lung and liver but not in kidney DNA, while DNA methylation in these organs remained unchanged [109]. Interestingly, a longitudinal panel study enrolling 36 healthy college students in Shanghai, China, showed a decrease in methylation of *angiotensin converting enzyme* (*ACE*) and an increase in blood ACE levels concomitant with elevated blood pressure upon short-term exposure to PM_2.5_ [110]. In mice exposed to PM_2.5_ through intratracheal instillation, an increase of pulmonary ACE production is accompanied by elevated angiotensin converting enzyme 2 (ACE2) level [111]. ACE2 is used for host cell entry by severe acute respiratory syndrome coronaviruses (SARS-CoVs) including severe acute respiratory syndrome coronavirus 2 (SARS-CoV-2) [112,113] detected in Wuhan, China, in December 2019 and causing coronavirus disease 2019 (COVID-19). Recent studies demonstrated that *ACE2* is epigenetically regulated and characterized by age and gender difference in DNA methylation in the respiratory system [114]. Aberrant hypomethylation and overexpression of *ACE2* in lupus patients may increase their susceptibility to SARS-CoV-2 infection and severity of COVID-19 [115]. Although direct evidence of 5hmC involvement into *ACE2* regulation by environmental and other factors is to be found, the existing data strongly suggest that DNA methylation/demethylation control of the *ACE2* gene, especially in persons exposed to certain hazards, should be considered as a possible approach for COVID-19 prevention and treatment.

PM_10_ exposure causes a decrease in 5mC and an increase in 5hmC levels in human peripheral blood mononuclear cells in vitro [116]. In office workers and truck drivers, ambient PM_10_ exposure is positively correlated with blood 5hmC, but not 5mC—most probably due to the generation of reactive oxygen species, which can stimulate oxidation of 5mC into 5hmC [117]. In contrast, buccal cells demonstrate a decrease both in 5hmC and 5mC upon exposure to ambient PM_2.5_ and PM_10_ levels [118]. The aforementioned data indicate that particulate air pollution has significant genome-wide and gene-specific epigenetic effects resulting in altered gene function and thus mediating development of different disorders.

#### 3.2.3. Bisphenol A

BPA or 4,4′-dihydroxy-2,2-diphenylpropane is one of the highest volume chemicals produced worldwide. BPA is a plastic monomer and plasticizer used in the production of polycarbonate plastics and epoxy resins, which are components of many consumer products including metal jar lids, food-contact surface lacquer coatings for cans, protective coatings and finishes, automobile parts, adhesives, food packaging, and plastic bottles. BPA is ubiquitous in the environment, as it is released from polycarbonate plastics and epoxy resins during sterilization, autoclaving, and re-heating. BPA is a toxin known to exert low estrogen activity [119]. In mice, high-dose BPA exposure increased fetal loss, whereas low-dose exposure can cause long-term disrupting effects on sexual differentiation, brain development, immune system, and behavior [120,121]. BPA exposure can have a transgenerational effect [122,123]. In humans, BPA exposure can induce diabetes mellitus, cardiovascular diseases, obesity, deterioration of sperm quality, and increased risk of reproductive losses [124,125,126,127]. 

A few papers report specific BPA-induced alterations in DNA hydroxymethylation patterns in human sperm cells. Workers in factories manufacturing BPA showed increased total 5hmC and LINE-1 hydroxymethylation levels in sperm cells [128,129]. Moreover, BPA-exposed individuals contained in sperm DNA 8670 hyper-hydroxymethylated regions and 940 hypo-hydroxymethylated regions affecting genes associated with the nervous system, development, cardiovascular diseases and signal transduction, some maternally expressed imprinted genes, and sperm-expressed genes, including *ACHE* gene [128,130]. 

Perinatal BPA exposure alters DNA hydroxymethylation patterns in 5950 regions, including 12 regions annotated to imprinted genes (*Gnas*, *Grb10*, *Plagl1*, *Klf14*, *Pde10a*, *Snrpn*, *Airn*, *Cmah*, *Ppp1r9a*, *Kcnq1*, *Phactr2*, and *Pde4d*) in mouse blood; these changes persist throughout adulthood, indicating longitudinal effects of BPA on 5hmC [131]. Perinatal BPA exposure increases *Kcnq1* expression in the brains of adult mice, as well as reprograms expression of epigenetic writers *Dnmt1* and *Tet2* [132]. However, 5hmC and 5mC enrichment in *Kcnq1* is not affected by BPA, suggesting that alterations in *Kcnq1*, *Dnmt1*, and *Tet2* expression are not linked to epigenetic changes in this locus [132]. In human estrogen-receptor positive breast cancer cells, BPA represses *TET2* expression, reduces TET2 protein production and decreases DNA hydroxymethylation, indicating the involvement of the epigenetic pathway in the BPA-mediated tumor cell proliferation [133].

#### 3.2.4. Hydroquinone

Hydroquinone is a metabolite of benzene—an environmental toxicant found in cigarette smoke and petroleum products. In HEK293T cells, hydroquinone exposure promotes the generation of reactive oxygen species and enhances TET1 activity with a decrease in global 5mC and an increase in global 5hmC [134].

#### 3.2.5. Pentachlorophenol metabolites

Pentachlorophenol (PCP) is widely used in wood protection as a bactericide, fungicide, molluscicide, herbicide, algaecide, and insecticide. PCP is resistant to degradation and persists in soil and water systems for up to several months. The International Agency for Research on Cancer classifies PCP as a B2 carcinogen (possibly carcinogenic to humans). Tetrachlorohydroquinone and tetrachloro-1,4-benzoquinone are two reactive metabolites of PCP playing a central role in its genotoxicity [135]. Both compounds are redox-active quinones that induce a 5hmC increase in lung adenocarcinoma (A549), HepG2, MRC5 cells, and mESCs [67,136]. The tetrachloro-1,4-benzoquinone-induced increase of hydroxymethylation in MRC5 cells affects 5751 genes and alters the expression in 3414 of them, including those related to the apoptosis signaling pathway [67]. The mechanism of action of quinones involves the ability to increase the cellular level of Fe(II) which stimulates the enzymatic activity of TET proteins, thus promoting the oxidation of 5mC to 5hmC [67,136].

The data on 5hmC changes upon exposure to the aforementioned hypnotics, medications, and anthropogenic pollutants are summarized in Table 1.

## 4. Conclusions and Future Perspectives

Although 5hmC has been known as one of the central components of the epigenetic network for over 10 years, our knowledge about molecular mechanisms underlying alterations in hydroxymethylation patterns and associated changes in mammalian cell gene expression in response to environmental factors is still in its early days. Accumulated research findings convincingly demonstrate that 5hmC is sensitive to environmental stimuli, and the associated alterations in 5hmC patterns can drive changes in gene expression, resulting in short- or long-term health effects. Most of such studies, however, are purely descriptive and merely establish a causal link between some environmental factor and alterations in DNA hydroxymethylation as a key finding, without shedding light on underlying molecular mechanisms. Rigorous studies on biochemical mechanisms of 5hmC modifications caused by environmental factors are scarce. They give trustworthy evidence that environmental factors in their ability to affect DNA hydroxymethylation directly or indirectly modify key components of TET-mediated oxidation of 5mC to 5hmC. Thus, different factors would apparently initiate different molecular mechanisms of epigenetic changes. 

This review provides data showing that modifications in 5hmC patterns induced by environmental factors most often involve TET-mediated active DNA demethylation. The latter induces increased DNA hydroxymethylation and decreased DNA methylation due to reactive oxygen species and iron(II) among other factors. Such a mechanism may be triggered in certain tissues/cell types by the exposure effect of phenobarbital, DMSO, particulate air pollution, PCP metabolites, and hydroquinone [67,69,70,91,116,134,136]. Decreased 5hmC levels can be associated with both TET down-regulation [73,92,96,97,104] and preceding reduction in 5mC—a substrate for oxidation [83,96,118]. In many cases, however, biochemical pathways of 5hmC alterations remain unknown. An example of a challenging case in point are elevated 5hmC and unchanged 5mC levels in visceral organs exposed to sodium arsenite [95]. A possible source for 5hmC here can be oxidation of de novo methylated cytosine as is the case for male pronucleus in mouse zygotes [137]. 

It is still unclear what mechanisms underlie the gene-specific impact of environmental factors on 5hmC. Studies investigating both global and gene-specific effects induced by environmental factors rigorously demonstrate that global increase/decrease in hydroxymethylation is accompanied by gene-specific 5hmC alterations—increased 5hmC in some genes and decreased 5hmC in other genes [67,78,84,131]—which in turn can differentially impact gene expression. Another curious fact to investigate is cell-, organ-, and tissue-specificity of 5hmC alterations upon exposure to the same environmental factor. To some extent, such specificity may come from metabolic properties of affecting environmental chemicals. This fact, as well as the observed sex difference in altered patterns of hydroxymethylation [101,102], demonstrates that changes in 5hmC levels are driven by a large set of metabolic pathways.

Another promising and compelling area to study refers to the effect of environmental factors on DNA hydroxymethylation during gametogenesis and embryogenesis, i.e., at stages when epigenetic genome reprogramming occurs [138,139,140,141]. At these particular stages, dynamic changes of 5hmC profiles determined by the developmental program may enhance susceptibility to environmental effects. In turn, environmentally induced epigenetic changes can have transgenerational effects in gametes and a long-term impact in adulthood in preimplantation embryos [142]. 

To conclude, alterations in DNA hydroxymethylation patterns can be regarded as a sensitive response indicator to many environmental factors. Underlying mechanisms and their impact on genome function differ in terms of environmental exposures that specifically target 5hmC in different organs, cell types, and DNA sequences. The ability of 5hmC patterns to undergo alterations in response to harmful environmental exposure undoubtedly presents a ‘weak link’ within the epigenome. It is epigenetic plasticity, however, which is based on the dynamic interplay between the regulatory effects of histone modifications and DNA methylation/hydroxymethylation, that apparently ensures genome flexibility and allows living organisms to adapt to the transforming environment. The sensitivity of DNA hydroxymethylation to environmental factors provides the possibility of purposefully changing 5hmC patterns by different effectors. Once impaired, a recovery to normal DNA hydroxymethylation could be attempted through modifying the availability of components for DNA methylation/hydroxymethylation. This may imply an appropriate SAM, Fe(II), and ascorbate supplementation as well as antioxidant therapy to reduce epigenetic consequences of oxidative stress. Such approaches are going to give opportunities to prevent or ameliorate different pathological conditions that strike residents living in contaminated areas or those exposed to occupational hazards, anticipate potential epigenetic transgenerational effects, and ensure better safety for future generations. Endeavors to develop effective epigenetic therapies and chemoprevention of environmentally induced epigenetic toxicity involving 5hmC patterns require a thorough understanding of molecular mechanisms underlying alterations in DNA hydroxymethylation to empower further rigorous investigation.

## Figures and Tables

**Table 1:** 5-hydroxymethylcytosine changes in mammalian genome upon exposure to external factors.

External Factor		Species	Condition	Organ/Tissue/Cell Line	Genomic Region	5hmC Alteration	Ref.
Hypnotics and Medications							
Phenobarbital		Mouse	In vivo	Liver	Upstream, promoter, and gene body regions of multiple genes from *Cyp2b* and *2c* families	Increase	[69]
					Multiple genes	Differential DNA hydroxymethylation	[70]
				Phenobarbital-induced hepatocellular adenoma	Multiple genes	Differential DNA hydroxymethylation	[71]
Diethylstilbestrol		Mouse	In vivo	Uterus	Genomic DNA	Decrease	[73]
Cocaine		Mouse	In vivo	Liver	Genomic DNA	Decrease	[77]
				Brain	Genomic DNA	No change	[77]
				Brain (nucleus accumbens)	Genomic DNA	No change	[78]
					Multiple genes	Differential DNA hydroxymethylation	[78]
		Rat	In vivo	Brain (prefrontal cortex)	Promoter of *Homer2* gene	Decrease	[79]
Methamphetamine		Rat	In vivo	Brain (striatum)	Promoters of *GluA1* and *GluA2* genes	Decrease	[83]
				Brain (nucleus accumbens)	Transcription start site of *Crh* gene; intragenic sites of *Avp* gene	Increase	[82]
					Multiple genes	Differential DNA hydroxymethylation	[84]
Ethanol		Human	In vivo	Blood	Genomic DNA	Decrease during consumption; Increase after detoxification	[88]
				Liver	Genomic DNA	Decrease	[90]
		Rat	In vivo	Liver	Genomic DNA	Decrease	[90]
					Genomic DNA	Decrease	[89]
		Mouse	In vivo	Brain (hippocampus)	Genomic DNA	Decrease	[85]
				Brain (cortex: cortical plate)	Genomic DNA	Increase	[86]
				Brain (cortex: subplate)	Genomic DNA	Decrease	[86]
				Brain (cortex: subventricular zone/ventricular zone)	Genomic DNA	Decrease	[86]
			In vitro	Forebrains neural stem cells	Promoters R1, R2, R3, R5 of *MeCP2* gene	Increase	[87]
					Genomic DNA	No change during exposure, Decrease after withdrawal	[87]
Dimethyl sulfoxide		Mouse	In vitro	MC3T3-E1	Genomic DNA	Short-term increase	[91]
					Promoters of *Fas* and *Dlx5* genes	Short-term increase	[91]
*Anthropogenic pollutants*							
Heavy metals	Arsenic	Human	In vivo	Blood	Genomic DNA	Decrease	[100]
				Blood	Genomic DNA	Increase (males); Decrease (females)	[101]
			In vitro	HepG2	Genomic DNA	Increase	[99]
				HEK293T	Genomic DNA	Decrease	[98]
		Rat	In vivo	Brain (cortex)	Genomic DNA	Decrease	[96]
				Brain (Hippocampus)	Genomic DNA	Decrease	[96]
				Heart	Genomic DNA	Increase	[95]
				Spleen	Genomic DNA	Increase	[95]
				Lung	Genomic DNA	Increase	[95]
				Pancreas	Genomic DNA	Decrease	[95]
				Liver	Genomic DNA	No changes	[95]
				Kidney	Genomic DNA	No changes	[95]
		Mouse	In vitro	mESCs	Genomic DNA	Decrease	[98]
					Genomic DNA	Decrease	[97]
	Mercury	Human	In vivo	Blood	Genomic DNA	Decrease	[103]
	Nickel	Human	In vitro	HEK293T	Genomic DNA	Decrease	[104]
				MRC5	Genomic DNA	Decrease	[104]
		Mouse	In vitro	mESCs	Genomic DNA	Decrease	[104]
	Cadmium	Human	In vivo	Blood	Genomic DNA	Increase (males); No changes (females)	[102]
		Mouse	In vitro	mESCs	Genomic DNA	Decrease	[97]
	Chromium	Human	In vivo	Blood	Genomic DNA	No change	[102]
		Mouse	In vitro	mESCs	Genomic DNA	Decrease	[97]
	Antimony	Mouse	In vitro	mESCs	Genomic DNA	Decrease	[97]
	Lead	Human	In vivo	Umbilical cord blood	Transcription start sites of *GSTM1* and *GSTM5* genes; Imprinted loci *PEG10*, *SGCE*	Decrease	[105]
				Blood	Genomic DNA	No change	[102]
			In vitro	hESCs	Transcription start sites of *GSTM1* and *GSTM5* genes; Imprinted loci *PEG10*, *SGCE*	Decrease	[105]
Particulate air pollution	PM_2.5_	Human	In vivo	Buccal cells	Genomic DNA	Decrease	[118]
			In vitro	SH-SY5Y	Genomic DNA	Increase	[108]
					Promoters of *MeCP2*, *GRIN1*, *GABRB3*, *NRXN1*, *NLGN3* genes	Increase	[108]
		Mouse	In vivo	Lung	Genomic DNA	Decrease	[109]
				Liver	Genomic DNA	Decrease	[109]
				Kidney	Genomic DNA	No change	[109]
	PM_10_	Human	In vivo	Blood	Genomic DNA	Increase	[117]
				Buccal cells	Genomic DNA	Decrease	[118]
			In vitro	Blood	Genomic DNA	Increase	[116]
Bisphenol A		Human	In vivo	Sperm	LINE1	Increase	[129]
						Increase	[128]
					Genomic DNA	Increase	[128]
					*ACHE* gene	Increase	[130]
			In vitro	MCF-7	Genomic DNA	Decrease	[133]
		Mouse	In vivo	Brain (cortex)	*Kcnq1* locus	No change	[132]
				Brain (midbrain)	*Kcnq1* locus	No change	[132]
				Blood	*Gnas, Grb10, Plagl1, Pde10a, Pde4d* genes	Increase	[131]
					*Klf14,Airn, Cmah, Snrpn, Ppp1r9a, Kcnq1, Phactr2* genes	Decrease	[131]
Hydroquinone		Human	In vitro	HEK293	Genomic DNA	Increase	[134]
					Open reading frame 2 of LINE1	Decrease	[134]
					Promoters of *GCLC* and *14-3-3σ* genes	Increase	[134]
Pentachlorophenol metabolites	Tetrachloro-1,4-benzoquinone	Human	In vitro	A549	Genomic DNA	Increase	[67]
				HepG2	Genomic DNA	Increase	[67]
				MRC5	Genomic DNA	Increase	[67]
		Mouse	In vitro	mESCs	Genomic DNA	Increase	[136]
	Tetrachloro-1,4-hydroquinone	Human	In vitro	A549	Genomic DNA	Increase	[67]
				HepG2	Genomic DNA	Increase	[67]
				MRC5	Genomic DNA	Increase	[67]

## Abbreviations

5caC	5-carboxylcytosine
5fC	5-formylcytosine
5hmC	5-hydroxymethylcytosine
5mC	5-methylcytosine
A549	human Caucasian lung carcinoma cell line
ACE	angiotensin converting enzyme
ACE2	angiotensin converting enzyme 2
AMPAR	alpha-amino-3-hydroxy-5-methyl-4-isoxazole propionic acid receptor
*Avp*	arginine vasopressin
BPA	bisphenol A
CAR	constitutive androstane receptor
COVID-19	coronavirus disease 2019
*Crh*/*Crf*	corticotropin-releasing hormone
DMSO	dimethyl sulfoxide
DNMTs	DNA methyltransferases
HEK293T	human embryonic kidney cell line
hESCs	human embryonic stem cells
HepG2	human Caucasian hepatocyte carcinoma cell line
HIF	hypoxia-inducible factor
IDHs	isocitrate dehydrogenases human
MC3T3-E1	mouse osteoblastic cell line
MCF-7	human breast cancer cell line
mESCs	mouse embryonic stem cells
MRC5	human embryonic lung fibroblasts cell culture (medical research council cell strain 5)
PCP	pentachlorophenol
SAM	S-adenosylmethionine
SARS-CoV	severe acute respiratory syndrome coronavirus
SARS-CoV-2	severe acute respiratory syndrome coronavirus 2
SH-SY5Y	human neuroblastoma cell line
TETs	Ten-Eleven Translocation enzymes

## References

[1] Penn N.W., Suwalski R., O’riley C., Bojanowski K., Yura R. (1972). The presence of 5-hydroxymethylcytosine in animal deoxyribonucleic acid. Biochem. J..

[2] Kriaucionis S., Heintz N. (2009). The nuclear DNA base 5-hydroxymethylcytosine is present in Purkinje neurons and the brain. Science.

[3] Tahiliani M., Koh K.P., Shen Y., Pastor W.A., Bandukwala H., Brudno Y., Agarwal S., Iyer L.M., Liu D.R., Aravind L. (2009). Conversion of 5-methylcytosine to 5-hydroxymethylcytosine in mammalian DNA by MLL partner TET1. Science.

[4] Santos F., Dean W. (2004). Epigenetic reprogramming during early development in mammals. Reproduction.

[5] Pendina A.A., Efimova O.A., Fedorova I.D., Leont’eva O.A., Shilnikova E.M., Lezhnina J.G., Kuznetzova T.V., Baranov V.S. (2011). DNA methylation patterns of metaphase chromosomes in human preimplantation embryos. Cytogenet. Genome Res..

[6] Jones P.A. (2012). Functions of DNA methylation: Islands, start sites, gene bodies and beyond. Nat. Rev. Genet..

[7] Vasilyev S.A., Tolmacheva E.N., Lebedev I.N. (2016). Epigenetic regulation and role of LINE-1 retrotransposon in embryogenesis. Russ. J. Genet..

[8] Ito S., D’Alessio A.C., Taranova O.V., Hong K., Sowers L.C., Zhang Y. (2010). Role of Tet proteins in 5mC to 5hmC conversion, ES-cell self-renewal and inner cell mass specification. Nature.

[9] Iurlaro M., Ficz G., Oxley D., Raiber E.A., Bachman M., Booth M.J., Andrews S., Balasubramanian S., Reik W. (2013). A screen for hydroxymethylcytosine and formylcytosine binding proteins suggests functions in transcription and chromatin regulation. Genome Biol..

[10] Spruijt C.G., Gnerlich F., Smits A.H., Pfaffeneder T., Jansen P.W., Bauer C., Eberl H.C. (2013). Dynamic readers for 5-(hydroxy) methylcytosine and its oxidized derivatives. Cell.

[11] Zhou T., Xiong J., Wang M., Yang N., Wong J., Zhu B., Xu R.M. (2014). Structural basis for hydroxymethylcytosine recognition by the SRA domain of UHRF2. Mol. Cell.

[12] Stroud H., Feng S., Kinney S.M., Pradhan S., Jacobsen S.E. (2011). 5-Hydroxymethylcytosine is associated with enhancers and gene bodies in human embryonic stem cells. Genome Biol..

[13] Hon G.C., Song C.X., Du T., Jin F., Selvaraj S., Lee A.Y., Kuan S. (2014). 5mC oxidation by Tet2 modulates enhancer activity and timing of transcriptome reprogramming during differentiation. Mol. Cell.

[14] Lu F., Liu Y., Jiang L., Yamaguchi S., Zhang Y. (2014). Role of Tet proteins in enhancer activity and telomere elongation. Genes Dev..

[15] Williams K., Christensen J., Helin K. (2012). DNA methylation: TET proteins—Guardians of CpG islands?. EMBO Rep..

[16] Song J., Pfeifer G.P. (2016). Are there specific readers of oxidized 5-methylcytosine bases?. Bioessays.

[17] Branco M.R., Ficz G., Reik W. (2011). Uncovering the role of 5-hydroxymethylcytosine in the epigenome. Nat. Rev. Genet..

[18] Efimova O.A., Pendina A.A., Tikhonov A.V., Kuznetzova T.V., Baranov V.S. (2015). Oxidized form of 5-methylcytosine—5-hydroxymethylcytosine: A new insight into the biological significance in the mammalian genome. Russ. J. Genet. Appl. Res..

[19] Efimova O.A., Pendina A.A., Tikhonov A.V., Baranov V.S. (2018). The evolution of ideas on the biological role of 5-methylcytosine oxidative derivatives in the mammalian genome. Russ. J. Genet. Appl. Res..

[20] Kantidze O.L., Razin S.V. (2017). 5-hydroxymethylcytosine in DNA repair: A new player or a red herring?. Cell Cycle.

[21] Wu X., Zhang Y. (2017). TET-mediated active DNA demethylation: Mechanism, function and beyond. Nat. Rev. Genet..

[22] Cantone I., Fisher A.G. (2013). Epigenetic programming and reprogramming during development. Nat. Struct. Mol. Biol..

[23] Efimova O.A., Pendina A.A., Krapivin M.I., Kopat V.V., Tikhonov A.V., Petrovskaia-Kaminskaia A.V., Navodnikova P.M., Talantova O.E., Glotov O.S., Baranov V.S. (2018). Inter-Cell and Inter-Chromosome Variability of 5-Hydroxymethylcytosine Patterns in Noncultured Human Embryonic and Extraembryonic Cells. Cytogenet. Genome Res..

[24] Efimova O.A., Pendina A.A., Lezhnina Y.G., Tikhonov A.V., Chiryaeva O.G., Petrova L.I., Talantova O.E., Dudkina V.S., Koltsova A.S., Krapivin M.I. (2019). Study of acetylated histone H3K9—An active chromatin mark—In chromosomes from adult and fetal human lymphocytes. Ecol. Genet..

[25] Patkin E.L., Sofronov G.A. (2013). Population epigenetics, ecotoxicology, and human diseases. Russ. J. Genet. Appl. Res..

[26] Salemi R., Marconi A., Di Salvatore V., Franco S., Rapisarda V., Libra M. (2017). Epigenetic alterations and occupational exposure to benzene, fibers, and heavy metals associated with tumor development. Mol. Med. Rep..

[27] Skryabin N.A., Vasilyev S.A., Lebedev I.N. (2017). Epigenetic silencing of genomic structural variations. Russ. J. Genet..

[28] Martin E.M., Fry R.C. (2018). Environmental Influences on the Epigenome: Exposure- Associated DNA Methylation in Human Populations. Annu. Rev. Public Health.

[29] Koltsova A.S., Pendina A.A., Efimova O.A., Chiryaeva O.G., Kuznetzova T.V., Baranov V.S. (2019). On the Complexity of Mechanisms and Consequences of Chromothripsis: An Update. Front. Genet..

[30] Ingrosso D., Cimmino A., Perna A.F., Masella L., De Santo N.G., De Bonis M.L., Vacca M., D’Esposito M., D’Urso M., Galletti P. (2003). Folate treatment and unbalanced methylation and changes of allelic expression induced by hyperhomocysteinaemia in patients with uraemia. Lancet.

[31] Brocato J., Costa M. (2013). Basic mechanics of DNA methylation and the unique landscape of the DNA methylome in metal-induced carcinogenesis. Crit. Rev. Toxicol..

[32] Li S., Washburn K.A., Moore R., Uno T., Teng C., Newbold R.R., McLachlan J.A., Negishi M. (1997). Developmental exposure to diethylstilbestrol elicits demethylation of estrogen-responsive lactoferrin gene in mouse uterus. Cancer Res..

[33] Alworth L.C., Howdeshell K.L., Ruhlen R.L., Day J.K., Lubahn D.B., Huang T.H., Besch-Williford C.L., Vom Saal F.S. (2002). Uterine responsiveness to estradiol and DNA methylation are altered by fetal exposure to diethylstilbestrol and methoxychlor in CD-1 mice: Effects of low versus high doses. Toxicol. Appl. Pharmacol..

[34] Sato K., Fukata H., Kogo Y., Ohgane J., Shiota K., Mori C. (2008). Neonatal exposure to diethylstilbestrol alters expression of DNA methyltransferases and methylation of genomic DNA in the mouse uterus. Endocr. J..

[35] Gilbert K.M., Blossom S.J., Erickson S.W., Reisfeld B., Zurlinden T.J., Broadfoot B., West K., Bai S., Cooney C.A. (2016). Chronic exposure to water pollutant trichloroethylene increased epigenetic drift in CD4(+) T cells. Epigenomics.

[36] Baccarelli A., Bollati V. (2009). Epigenetics and environmental chemicals. Curr. Opin. Pediatr..

[37] Faulk C., Kim J.H., Anderson O.S., Nahar M.S., Jones T.R., Sartor M.A., Dolinoy D.C. (2016). Detection of differential DNA methylation in repetitive DNA of mice and humans perinatally exposed to bisphenol A. Epigenetics.

[38] Patkin E.L., Grudinina N.A., Sasina L.K., Noniashvili E.M., Pavlinova L.I., Suchkova I.O., Kustova M.E., Kolmakov N.N., Van Truong T., Sofronov G.A. (2017). Asymmetric DNA methylation between sister chromatids of metaphase chromosomes in mouse embryos upon bisphenol A action. Reprod. Toxicol..

[39] Noniashvili E.M., Grudinina N.A., Kustova M.E., Suchkova I.O., Pavlinova L.I., Sasina L.K., Patkin E.L. (2017). DNA methylation in early mice embryogenesis under the influence of bisphenol A. Ecol. Genet..

[40] Suchkova I.O., Sasina L.K., Dergacheva N.I., Sofronov G.A., Patkin E.L. (2019). The influence of low dose Bisphenol A on whole genome DNA methylation and chromatin compaction in different human cell lines. Toxicol. Vitr..

[41] Wang Z., Yang C. (2019). Metal carcinogen exposure induces cancer stem cell-like property through epigenetic reprograming: A novel mechanism of metal carcinogenesis. Semin. Cancer Biol..

[42] Suvorov A., Naumov V., Shtratnikova V., Logacheva M., Shershebnev A., Wu H., Gerasimov E., Zheludkevich A., Pilsner J.R., Sergeyev O. (2020). Rat liver epigenome programing by perinatal exposure to 2,2’,4’4’-tetrabromodiphenyl ether. Epigenomics.

[43] Dao T., Cheng R.Y., Revelo M.P., Mitzner W., Tang W. (2014). Hydroxymethylation as a Novel Environmental Biosensor. Curr. Environ. Health Rep..

[44] Kochmanski J., Bernstein A.I. (2020). The Impact of Environmental Factors on 5-Hydroxymethylcytosine in the Brain. Curr. Environ. Health Rep..

[45] Pulczinski J., Yeung B.H., Wu Q., Cheng R.Y., Tang W.Y., McCullough S.D., Dolinoy D.C. (2018). DNA Hydroxymethylation: Implications for Toxicology and Epigenetic Epidemiology. Toxicoepigenetics: Core Principles and Applications.

[46] Castro G.D., Díaz Gómez M.I., Castro J.A. (1996). 5-Methylcytosine attack by hydroxyl free radicals and during carbon tetrachloride promoted liver microsomal lipid peroxidation: Structure of reaction products. Chem. Biol. Interact..

[47] Madugundu G.S., Cadet J., Wagner J.R. (2014). Hydroxyl-radical-induced oxidation of 5-methylcytosine in isolated and cellular DNA. Nucleic Acids Res..

[48] Cadet J., Wagner J.R. (2016). Radiation-induced damage to cellular DNA: Chemical nature and mechanisms of lesion formation. Radiat. Phys. Chem..

[49] Cheng X., Kumar S., Posfai J., Pflugrath J.W., Roberts R.J. (1993). Crystal structure of the Hhal DNA methyltransferase complexed with S-adenosyl-L-methionine. Cell.

[50] Okano M., Xie S., Li E. (1998). Cloning and characterization of a family of novel mammalian DNA (cytosine-5) methyltransferases. Nat. Genet..

[51] Krebs H.A., Johnson W.A. (1937). The role of citric acid in intermediate metabolism in animal tissues. Enzymologia.

[52] Chou N.H., Tsai C.Y., Tu Y.T., Wang K.C., Kang C.H., Chang P.M., Li G.C., Lam H.C., Liu S.I., Tsai K.W. (2016). Isocitrate Dehydrogenase 2 Dysfunction Contributes to 5-hydroxymethylcytosine Depletion in Gastric Cancer Cells. Anticancer Res..

[53] Lian C.G., Xu Y., Ceol C., Wu F., Larson A., Dresser K., Xu W., Tan L., Hu Y., Zhan Q. (2012). Loss of 5-hydroxymethylcytosine is an epigenetic hallmark of melanoma. Cell.

[54] Xu W., Yang H., Liu Y., Yang Y., Wang P., Kim S.H., Ito S., Yang C., Wang P., Xiao M.T. (2011). Oncometabolite 2-hydroxyglutarate is a competitive inhibitor of α-ketoglutarate-dependent dioxygenases. Cancer Cell.

[55] Xiao M., Yang H., Xu W., Ma S., Lin H., Zhu H., Zhao S. (2012). Inhibition of alpha--KG--dependent histone and DNA demethylases by fumarate and succinate that are accumulated in mutations of FH and SDH tumor suppressors. Genes Dev..

[56] Mason E.F., Hornick J.L. (2013). Succinate dehydrogenase deficiency is associated with decreased 5-hydroxymethylcytosine production in gastrointestinal stromal tumors: Implications for mechanisms of tumorigenesis. Mod. Pathol..

[57] Yang H., Lin H., Xu H., Zhang L., Cheng L., Wen B., Shou J., Guan K., Xiong Y., Ye D. (2014). TET-catalyzed 5-methylcytosine hydroxylation is dynamically regulated by metabolites. Cell Res..

[58] Burr S., Caldwell A., Chong M., Beretta M., Metcalf S., Hancock M., Arno M., Balu S., Kropf V.L., Mistry R.K. (2018). Oxygen gradients can determine epigenetic asymmetry and cellular differentiation via differential regulation of Tet activity in embryonic stem cells. Nucleic Acids Res..

[59] Thienpont B., Steinbacher J., Zhao H., D’Anna F., Kuchnio A., Ploumakis A., Hermans E. (2016). Tumour hypoxia causes DNA hypermethylation by reducing TET activity. Nature.

[60] Koutsouraki E., Pells S., De Sousa P.A. (2019). Sufficiency of hypoxia-inducible 2-oxoglutarate dioxygenases to block chemical oxidative stress-induced differentiation of human embryonic stem cells. Stem Cell Res..

[61] Mariani C.J., Vasanthakumar A., Madzo J., Yesilkanal A., Bhagat T., Yu Y., Verma A. (2014). TET1--mediated hydroxymethylation facilitates hypoxic gene induction in neuroblastoma. Cell Rep..

[62] Minor E.A., Court B.L., Young J.I., Wang G. (2013). Ascorbate induces ten-eleven translocation (Tet) methylcytosine dioxygenase-mediated generation of 5-hydroxymethylcytosine. J. Biol. Chem..

[63] Dickson K.M., Gustafson C.B., Young J.I., Züchner S., Wang G. (2013). Ascorbate-induced generation of 5-hydroxymethylcytosine is unaffected by varying levels of iron and 2-oxoglutarate. Biochem. Biophys. Res. Commun..

[64] Yin R., Mao S.Q., Zhao B., Chong Z., Yang Y., Zhao C., Zhang D., Huang H., Gao J., Li Z. (2013). Ascorbic Acid Enhances Tet-Mediated 5-Methylcytosine Oxidation and Promotes DNA Demethylation in Mammals. J. Am. Chem. Soc..

[65] Li W., Liu M. (2011). Distribution of 5-hydroxymethylcytosine in different human tissues. J. Nucleic. Acids.

[66] Gustafson C.B., Yang C., Dickson K.M., Shao H., Van Booven D., Harbour J.W., Wang G. (2015). Epigenetic reprogramming of melanoma cells by vitamin C treatment. Clin. Epigenetics.

[67] Zhao B., Yang Y., Wang X., Chong Z., Yin R., Song S.H., Zhao C., Li C., Huang H., Sun B.F. (2014). Redox-active quinones induces genome-wide DNA methylation changes by an iron-mediated and Tet-dependent mechanism. Nucleic Acids Res..

[68] Rice J.M., Diwan B.A., Hu H., Ward J.M., Nims R.W., Lubet R.A. (1994). Enhancement of hepatocarcinogenesis and induction of specific cytochrome P450-dependent monooxygenase activities by the barbiturates allobarbital, aprobarbital, pentobarbital, secobarbital and 5-phenyl-and 5-ethylbarbituric acids. Carcinogenesis.

[69] Thomson J.P., Lempiainen H., Tefferi A., Nestor C.E., Muller A., Bolognani F., Oakeley E.J., Schubeler D., Terranova R., Reinhardt D. (2012). Non-genotoxic carcinogen exposure induces defined changes in the 5-hydroxymethylome. Genome Biol..

[70] Thomson J.P., Hunter J.M., Lempiainen H., Muller A., Terranova R., Moggs J.G., Meehan R.R. (2013). Dynamic changes in 5-hydroxymethylation signatures underpin early and late events in drug exposed liver. Nucleic Acids Res..

[71] Ohara A., Takahashi Y., Kondo M., Okuda Y., Takeda S., Kushida M., Kobayashi K., Sumida K., Yamada T. (2017). Candidate genes responsible for early key events of phenobarbital-promoted mouse hepatocellular tumorigenesis based on differentiation of regulating genes between wild type mice and humanized chimeric mice. Toxicol. Res..

[72] Herbst A.L., Ulfelder H., Poskanzer D.C. (1971). Adenocarcinoma of the vagina: Association of maternal stilbestrol therapy with tumor appearance in young women. N. Engl. J. Med..

[73] Jefferson W.N., Chevalier D.M., Phelps J.Y., Cantor A.M., Padilla-Banks E., Newbold R.R., Archer T.K., Kinyamu H.K., Williams C.J. (2013). Persistently altered epigenetic marks in the mouse uterus after neonatal estrogen exposure. Mol. Endocrinol..

[74] Mahalingaiah S., Hart J.E., Wise L.A., Terry K.L., Boynton-Jarrett R., Missmer S.A. (2014). Prenatal diethylstilbestrol exposure and risk of uterine leiomyomata in the Nurses’ Health Study II. Am. J. Epidemiol..

[75] Navarro A., Yin P., Ono M., Monsivais D., Moravek M.B., Coon J.S., Dyson M.T., Wei J.J., Bulun S.E. (2014). 5-Hydroxymethylcytosine promotes proliferation of human uterine leiomyoma: A biological link to a new epigenetic modification in benign tumors. J. Clin. Endocrinol. Metab..

[76] Kol’tsova A.S., Pendina A.A., Efimova O.A., Kaminskaya A.N., Tikhonov A.V., Osinovskaya N.S., Sultanov I.Y., Shved N.Y., Kakhiani M.I., Baranov V.S. (2017). Differential DNA Hydroxymethylation in Human Uterine Leiomyoma Cells Depending on the Phase of Menstrual Cycle and Presence of MED12 Gene Mutations. Bull. Exp. Biol. Med..

[77] Chao M.R., Fragou D., Zanos P., Hu C.W., Bailey A., Kouidou S., Kovatsi L. (2014). Epigenetically modified nucleotides in chronic heroin and cocaine treated mice. Toxicol. Lett..

[78] Feng J., Shao N., Szulwach K.E., Vialou V., Huynh J., Zhong C., Le T., Ferguson D., Cahill M.E., Li Y. (2015). Role of Tet1 and 5 hydroxymethylcytosine in cocaine action. Nat. Neurosci..

[79] Ploense K.L., Li X., Baker-Andresen D., Carr A.E., Woodward N., Bagley J., Szumlinski K.K., Bredy T.W., Kippin T.E. (2018). Prolonged-access to cocaine induces distinct Homer2 DNA methylation, hydroxymethylation, and transcriptional profiles in the dorsomedial prefrontal cortex of Male Sprague-Dawley rats. Neuropharmacology.

[80] Saad L., Sartori M., Pol Bodetto S., Romieu P., Kalsbeek A., Zwiller J., Anglard P. (2019). Regulation of Brain DNA Methylation Factors and of the Orexinergic System by Cocaine and Food Self-Administration. Mol. Neurobiol..

[81] González B., Pantoja C.R.G., Sosa M.H., Vitullo A.D., Bisagno V., González C.R. (2018). Cocaine alters the mouse testicular epigenome with direct impact on histone acetylation and DNA methylation marks. Reprod. Biomed. Online.

[82] Jayanthi S., Gonzalez B., McCoy M.T., Ladenheim B., Bisagno V., Cadet J.L. (2018). Methamphetamine Induces TET1- and TET3-Dependent DNA Hydroxymethylation of Crh and Avp Genes in the Rat Nucleus Accumbens. Mol. Neurobiol..

[83] Jayanthi S., McCoy M.T., Chen B., Britt J.P., Kourrich S., Yau H.J., Ladenheim B., Krasnova I.N., Bonci A., Cadet J.L. (2014). Methamphetamine downregulates striatal glutamate receptors via diverse epigenetic mechanisms. Biol. Psychiatry.

[84] Cadet J.L., Brannock C., Krasnova I.N., Jayanthi S., Ladenheim B., McCoy M.T., Walther D., Godino A., Pirooznia M., Lee R.S. (2017). Genome-wide DNA hydroxymethylation identifies potassium channels in the nucleus accumbens as discriminators of methamphetamine addiction and abstinence. Mol. Psychiatry.

[85] Chen Y., Ozturk N.C., Zhou F.C. (2013). DNA methylation program in developing hippocampus and its alteration by alcohol. PLoS ONE.

[86] Öztürk N.C., Resendiz M., Öztürk H., Zhou F.C. (2017). DNA Methylation program in normal and alcohol-induced thinning cortex. Alcohol.

[87] Liyanage V.R., Zachariah R.M., Davie J.R., Rastegar M. (2015). Ethanol deregulates Mecp2/MeCP2 in differentiating neural stem cells via interplay between 5-methylcytosine and 5-hydroxymethylcytosine at the Mecp2 regulatory elements. Exp. Neurol..

[88] Koller G., Zill P., Soyka M., Adorjan K., Weiss C., Kern A., Nguyen-Thien M.L., Kamp F., Proebstl L., Krause D. (2019). Short-term changes in global methylation and hydroxymethylation during alcohol detoxification. Eur. Neuropsychopharmacol..

[89] Tammen S.A., Park J.E., Shin P.K., Friso S., Chung J., Choi S.W. (2016). Iron Supplementation Reverses the Reduction of Hydroxymethylcytosine in Hepatic DNA Associated with Chronic Alcohol Consumption in Rats. J. Cancer. Prev..

[90] Ji C., Nagaoka K., Zou J., Casulli S., Lu S., Cao K.Y., Zhang H., Iwagami Y., Carlson R.I., Brooks K. (2019). Chronic ethanol mediated hepatocyte apoptosis links to decreased TET1 and 5-hydroxymethylcytosine formation. FASEB J..

[91] Thaler R., Spitzer S., Karlic H., Klaushofer K., Varga F. (2012). DMSO is a strong inducer of DNA hydroxymethylation in pre-osteoblastic MC3T3-E1 cells. Epigenetics.

[92] Verheijen M., Lienhard M., Schrooders Y., Clayton O., Nudischer R., Boerno S., Timmermann B., Selevsek N., Schlapbach R., Gmuender H. (2019). DMSO induces drastic changes in human cellular processes and epigenetic landscape in vitro. Sci. Rep..

[93] Hong Y.S., Song K.H., Chung J.Y. (2014). Health effects of chronic arsenic exposure. J. Prev. Med. Public Health.

[94] Ehrlich M. (2002). DNA methylation in cancer: Too much, but also too little. Oncogene.

[95] Zhang J., Mu X., Xu W., Martin F.L., Alamdar A., Liu L., Tian M., Huang Q., Shen H. (2014). Exposure to arsenic via drinking water induces 5-hydroxymethylcytosine alteration in rat. Sci. Total Environ..

[96] Du X., Tian M., Wang X., Zhang J., Huang Q., Liu L., Shen H. (2018). Cortex and hippocampus DNA epigenetic response to a long-term arsenic exposure via drinking water. Environ. Pollut..

[97] Xiong J., Liu X., Cheng Q.Y., Xiao S., Xia L.X., Yuan B.F., Feng Y.Q. (2017). Heavy Metals Induce Decline of Derivatives of 5-Methycytosine in Both DNA and RNA of Stem Cells. ACS Chem. Biol..

[98] Liu S., Jiang J., Li L., Amato N.J., Wang Z., Wang Y. (2015). Arsenite Targets the Zinc Finger Domains of Tet Proteins and Inhibits Tet-Mediated Oxidation of 5-Methylcytosine. Environ. Sci. Technol..

[99] Müller S.M., Finke H., Ebert F., Kopp J.F., Schumacher F., Kleuser B., Francesconi K.A., Raber G., Schwerdtle T. (2018). Arsenic-containing hydrocarbons: Effects on gene expression, epigenetics, and biotransformation in HepG2 cells. Arch. Toxicol..

[100] Tellez-Plaza M., Tang W.Y., Shang Y., Umans J.G., Francesconi K.A., Goessler W., Ledesma M., Leon M., Laclaustra M., Pollak J. (2014). Association of global DNA methylation and global DNA hydroxymethylation with metals and other exposures in human blood DNA samples. Environ. Health Perspect..

[101] Niedzwiecki M.M., Liu X., Hall M.N., Thomas T., Slavkovich V., Ilievski V., Levy D., Alam S., Siddique A.B., Parvez F. (2015). Sex-specific associations of arsenic exposure with global DNA methylation and hydroxymethylation in leukocytes: Results from two studies in Bangladesh. Cancer Epidemiol. Biomark. Prev..

[102] Xu P., Chen Z., Chen Y., Feng L., Wu L., Xu D., Wang X., Lou X., Lou J. (2019). Body burdens of heavy metals associated with epigenetic damage in children living in the vicinity of a municipal waste incinerator. Chemosphere.

[103] Cardenas A., Rifas-Shiman S.L., Godderis L., Duca R.C., Navas-Acien A., Litonjua A.A., DeMeo D.L., Brennan K.J., Amarasiriwardena C.J., Hivert M.F. (2017). Prenatal Exposure to Mercury: Associations with Global DNA Methylation and Hydroxymethylation in Cord Blood and in Childhood. Environ. Health Perspect..

[104] Yin R., Mo J., Dai J., Wang H. (2018). Nickel(ii) inhibits the oxidation of DNA 5-methylcytosine in mammalian somatic cells and embryonic stem cells. Metallomics.

[105] Sen A., Cingolani P., Senut M.C., Land S., Mercado-Garcia A., Tellez-Rojo M.M., Baccarelli A.A., Wright R.O., Ruden D.M. (2015). Lead exposure induces changes in 5-hydroxymethylcytosine clusters in CpG islands in human embryonic stem cells and umbilical cord blood. Epigenetics.

[106] Heusinkveld H.J., Wahle T., Campbell A., Westerink R.H.S., Tran L., Johnston H., Stone V., Cassee F.R., Schins R.P.F. (2016). Neurodegenerative and neurological disorders by small inhaled particles. Neurotoxicology.

[107] Calderón-Garcidueñas L., Leray E., Heydarpour P., Torres-Jardón R., Reis J. (2016). Air pollution, a rising environmental risk factor for cognition, neuroinflammation and neurodegeneration: The clinical impact on children and beyond. Rev. Neurol..

[108] Wei H., Feng Y., Liang F., Cheng W., Wu X., Zhou R., Wang Y. (2017). Role of oxidative stress and DNA hydroxymethylation in the neurotoxicity of fine particulate matter. Toxicology.

[109] De Oliveira A.A.F., De Oliveira T.F., Dias M.F., Medeiros M.H.G., Di Mascio P., Veras M., Lemos M., Marcourakis T., Saldiva P.H.N., Loureiro A.P.M. (2018). Genotoxic and epigenotoxic effects in mice exposed to concentrated ambient fine particulate matter (PM(2.5)) from São Paulo city, Brazil. Part. Fibre. Toxicol..

[110] Wang C., Chen R., Cai J., Shi J., Yang C., Tse L.A., Li H., Lin Z., Meng X., Liu C. (2016). Personal exposure to fine particulate matter and blood pressure: A role of angiotensin converting enzyme and its DNA methylation. Environ Int..

[111] Lin C.I., Tsai C.H., Sun Y.L., Hsieh W.Y., Lin Y.C., Chen C.Y., Lin C.S. (2018). Instillation of particulate matter 2.5 induced acute lung injury and attenuated the injury recovery in ACE2 knockout mice. Int. J. Biol. Sci..

[112] Li W., Moore M.J., Vasilieva N., Sui J., Wong S.K., Berne M.A., Somasundaran M., Sullivan J.L., Luzuriaga K., Greenough T.C. (2003). Angiotensin-converting enzyme 2 is a functional receptor for the SARS coronavirus. Nature.

[113] Hoffmann M., Kleine-Weber H., Schroeder S., Krüger N., Herrler T., Erichsen S., Schiergens T.S., Herrler G., Wu N.H., Nitsche A. (2020). SARS-CoV-2 Cell Entry Depends on ACE2 and TMPRSS2 and Is Blocked by a Clinically Proven Protease Inhibitor. Cell.

[114] Corley M.J., Ndhlovu L.C. (2020). DNA Methylation Analysis of the COVID-19 Host Cell Receptor, Angiotensin I Converting Enzyme 2 Gene (ACE2) in the Respiratory System Reveal Age and Gender Differences. Preprints.

[115] Sawalha A.H., Zhao M., Coit P., Lu Q. (2020). Epigenetic dysregulation of ACE2 and interferon-regulated genes might suggest increased COVID-19 susceptibility and severity in lupus patients. Clin. Immunol..

[116] Faraji M., Pourpak Z., Naddafi K., Nodehi R.N., Nicknam M.H., Shamsipour M., Rezaei S., Ghozikali M.G., Ghanbarian M., Mesdaghinia A. (2018). Effects of airborne particulate matter (PM10) from dust storm and thermal inversion on global DNA methylation in human peripheral blood mononuclear cells (PBMCs) in vitro. Atmos. Environ..

[117] Sanchez-Guerra M., Zheng Y., Osorio-Yanez C., Zhong J., Chervona Y., Wang S., Chang D., McCracken J.P., Díaz A., Bertazzi P.A. (2015). Effects of particulate matter exposure on blood 5-hydroxymethylation: Results from the Beijing truck driver air pollution study. Epigenetics.

[118] De Nys S., Duca R.C., Nawrot T., Hoet P., Van Meerbeek B., Van Landuyt K.L., Godderis L. (2018). Temporal variability of global DNA methylation and hydroxymethylation in buccal cells of healthy adults: Association with air pollution. Environ. Int..

[119] Laws S.C., Carey S.A., Ferrell J.M., Bodman G.J., Cooper R.L. (2000). Estrogenic activity of octylphenol, nonylphenol, bisphenol A and methoxychlor in rats. Toxicol. Sci..

[120] Gioiosa L., Fissore E., Ghirardelli G., Parmigiani S., Palanza P. (2007). Developmental exposure to low-dose estrogenic endocrine disruptors alters sex differences in exploration and emotional responses in mice. Horm. Behav..

[121] Koike E., Yanagisawa R., Win-Shwe T.T., Takano H. (2018). Exposure to low-dose bisphenol A during the juvenile period of development disrupts the immune system and aggravates allergic airway inflammation in mice. Int. J. Immunopathol. Pharmacol..

[122] Zhang X.F., Zhang L.J., Feng Y.N., Chen B., Feng Y.M., Liang G.J., Shen W. (2012). Bisphenol A exposure modifies DNA methylation of imprint genes in mouse fetal germ cells. Mol. Biol. Rep..

[123] Lombó M., Fernández-Díez C., González-Rojo S., Navarro C., Robles V., Herráez M.P. (2015). Transgenerational inheritance of heart disorders caused by paternal bisphenol A exposure. Environ. Pollut..

[124] Sugiura-Ogasawara M., Ozaki Y., Sonta S.I., Makino T., Suzumori K. (2005). Exposure to bisphenol A is associated with recurrent miscarriage. Hum. Reprod..

[125] Meeker J.D., Ehrlich S., Toth T.L., Wright D.L., Calafat A.M., Trisini A.T., Hauser R. (2010). Semen quality and sperm DNA damage in relation to urinary bisphenol A among men from an infertility clinic. Reprod. Toxicol..

[126] Bae S., Kim J.H., Lim Y.H., Park H.Y., Hong Y.C. (2012). Associations of bisphenol A exposure with heart rate variability and blood pressure. Hypertension.

[127] Gong H., Zhang X., Cheng B., Sun Y., Li C., Li T., Huang K. (2013). Bisphenol A accelerates toxic amyloid formation of human islet amyloid polypeptide: A possible link between bisphenol A exposure and type 2 diabetes. PLoS ONE.

[128] Zheng H., Zhou X., Li D.K., Yang F., Pan H., Li T., Miao M., Li R., Yuan W. (2017). Genome wide alteration in DNA hydroxymethylation in the sperm from bisphenol A exposed men. PLoS ONE..

[129] Tian Y., Zhou X., Miao M., Li D.K., Wang Z., Li R., Liang H., Yuan W. (2018). Association of Bisphenol A Exposure with LINE-1 Hydroxymethylation in Human Semen. Int. J. Environ. Res. Public Health.

[130] Song X., Miao M., Zhou X., Li D., Tian Y., Liang H., Li R., Yuan W. (2019). Bisphenol A Exposure and Sperm ACHE Hydroxymethylation in Men. Int. J. Environ. Res. Public Health.

[131] Kochmanski J.J., Marchlewicz E.H., Cavalcante R.G., Perera B.P.U., Sartor M.A., Dolinoy D.C. (2018). Longitudinal Effects of Developmental Bisphenol A Exposure on Epigenome Wide DNA Hydroxymethylation at Imprinted Loci in Mouse Blood. Environ. Health Perspect..

[132] Malloy M.A., Kochmanski J.J., Jones T.R., Colacino J.A., Goodrich J.M., Dolinoy D.C., Svoboda L.K. (2019). Perinatal Bisphenol A Exposure and Reprogramming of Imprinted Gene Expression in the Adult Mouse Brain. Front. Genet..

[133] Li Z., Lyu C., Ren Y., Wang H. (2020). Role of TET Dioxygenases and DNA Hydroxymethylation in Bisphenols-Stimulated Proliferation of Breast Cancer Cells. Environ. Health Perspect..

[134] Coulter J.B., O’Driscoll C.M., Bressler J.P. (2013). Hydroquinone increases 5-hydroxymethylcytosine formation through ten eleven translocation 1 (TET1) 5-methylcytosine dioxygenase. J. Biol. Chem..

[135] Schroeder I.E., Van Tonder J.J., Steenkamp V. (2012). Comparative toxicity of pentachlorophenol with its metabolites tetrachloro-1, 2-hydroquinone and tetrachloro-1, 4-benzoquinone in HepG2 cells. Open Toxicol. J..

[136] Li C., Wang F., Wang H. (2017). Tetrachloro-1,4-benzoquinone induces apoptosis of mouse embryonic stem cells. J. Environ. Sci..

[137] Amouroux R., Nashun B., Shirane K., Nakagawa S., Hill P.W., D’Souza Z., Encheva V. (2016). De novo DNA methylation drives 5hmC accumulation in mouse zygotes. Nat. Cell. Biol..

[138] Yamaguchi S., Hong K., Liu R., Inoue A., Shen L., Zhang K. (2013). Dynamics of 5-methylcytosine and 5-hydroxymethylcytosine during germ cell reprogramming. Cell Res..

[139] Efimova O.A., Pendina A.A., Tikhonov A.V., Fedorova I.D., Krapivin M.I., Chiryaeva O.G., Shilnikova E.M., Bogdanova M.A., Kogan I.Y., Kuznetzova T.V. (2015). Chromosome hydroxymethylation patterns in human zygotes and cleavage-stage embryos. Reproduction.

[140] Efimova O.A., Pendina A.A., Tikhonov A.V., Parfenyev S.E., Mekina I.D., Komarova E.M., Mazilina M.A., Daev E.V., Chiryaeva O.G., Galembo I.A. (2017). Genome-wide 5-hydroxymethylcytosine patterns in human spermatogenesis are associated with semen quality. Oncotarget.

[141] Pendina A.A., Efimova O.A., Krapivin M.I., Mekina I.D., Tikhonov A.V., Koltsova A.S., Petrovskaia-Kaminskaia A.V., Chiryaeva O.G., Kogan I.Y., Gzgzyan A.M. (2018). Genomic distribution of 5-formylcytosine and 5-carboxylcytosine in human preimplantation embryos. Mol. Reprod. Dev..

[142] Tikhodeyev O.N. (2020). Heredity determined by the environment: Lamarckian ideas in modern molecular biology. Sci. Total. Environ..

